# Modulation of endothelial organelle size as an antithrombotic strategy

**DOI:** 10.1111/jth.15084

**Published:** 2020-10-16

**Authors:** Francesco Ferraro, Francesca Patella, Joana R. Costa, Robin Ketteler, Janos Kriston‐Vizi, Daniel F. Cutler

**Affiliations:** ^1^ Endothelial Cell Biology Group, MRC Laboratory for Molecular Cell Biology University College London London UK; ^2^ Cell Signalling and Autophagy Group MRC Laboratory for Molecular Cell Biology University College London London UK; ^3^ Bioinformatics Image Core (BIONIC) MRC Laboratory for Molecular Cell Biology University College London London UK; ^4^ Present address: Department of Biology and Evolution of Marine Organisms (BEOM) Stazione Zoologica Anton Dohrn Villa Comunale Naples Italy; ^5^ Present address: Leukaemia Biology Research Group Department of Haematology, Cancer Institute University College London London UK

**Keywords:** COVID‐19, drug repurposing, thrombosis, von Willebrand factor, Weibel‐Palade bodies

## Abstract

**Background:**

It is long established that von Willebrand factor (VWF) is central to hemostasis and thrombosis. Endothelial VWF is stored in cell‐specific secretory granules, Weibel‐Palade bodies (WPBs), organelles generated in a wide range of lengths (0.5‐5.0 µm). WPB size responds to physiological cues and pharmacological treatment, and VWF secretion from shortened WPBs dramatically reduces platelet and plasma VWF adhesion to an endothelial surface.

**Objective:**

We hypothesized that WPB‐shortening represented a novel target for antithrombotic therapy. Our objective was to determine whether compounds exhibiting this activity do exist.

**Methods:**

Using a microscopy approach coupled to automated image analysis, we measured the size of WPB bodies in primary human endothelial cells treated with licensed compounds for 24 hours.

**Results and Conclusions:**

A novel approach to identification of antithrombotic compounds generated a significant number of candidates with the ability to shorten WPBs. In vitro assays of two selected compounds confirm that they inhibit the pro‐hemostatic activity of secreted VWF. This set of compounds acting at a very early stage of the hemostatic process could well prove to be a useful adjunct to current antithrombotic therapeutics. Further, in the current SARS‐CoV‐2 pandemic, with a considerable fraction of critically ill COVID‐19 patients affected by hypercoagulability, these WPB size‐reducing drugs might also provide welcome therapeutic leads for frontline clinicians and researchers.

**Essentials**

- von Willebrand factor (VWF) stored in Weibel‐Palade bodies (WPBs) is central to hemostasis and thrombosis.
- VWF secretion from shorter WPBs dramatically reduces primary hemostatic activities.
- Shortening WPBs thus provides the opportunity to reduce thrombotic risk.
- Screening licensed drugs for WPB‐shortening activity reveals new potential anti‐thrombotic compounds.

## 1 INTRODUCTION

Endothelial von Willebrand factor (VWF) plays a fundamental role in hemostasis, with deficiencies in its activity causing von Willebrand disease (VWD), the most common inherited human bleeding disorder.^1^ VWF is a large multi‐domain glycoprotein, whose function in hemostasis depends on its multimeric status. VWF multimers act as mechano‐transducers, which respond to shear forces in the circulation by stretching open and exposing binding sites for integrins, collagen, platelets, and homotypic interaction (ie, between VWF multimers).^2^ Endothelial cells secrete VWF in a highly multimerized form, known as ultra‐large (UL)‐VWF, extremely sensitive to hemodynamic forces and thus very active in platelet binding.^3^

UL‐VWF’s potential to cause spontaneous thrombus formation is controlled by a circulating protease, ADAMTS13, which generates the less‐multimerized, less active forms of VWF seen in plasma.^3^ Persistence of UL‐VWF in the circulation leads to microvascular thrombosis and the highly morbid and potentially life threatening clinical manifestations observed in a host of infectious and non‐infectious diseases, such as sepsis and thrombotic thrombocytopenic purpura (TTP).^4^, ^5^ Although these may be the most extreme examples of excess VWF function, many common disorders including hypertension and diabetes are characterized by increased VWF plasma levels.^6^, ^7^

While VWF is stored in the secretory granules of both platelets and endothelial cells, most of the VWF circulating in plasma derives from endothelial Weibel‐Palade bodies (WPBs) and is fundamental to hemostasis.^8^, ^9^ Vessel injury, in either the macro‐ or microcirculation, triggers localized stimulated exocytosis of WPBs, mediated by a variety of agonists.^10^ UL‐VWF secreted from activated endothelium forms cable‐like structures built of multiple multimers, both in vitro and in vivo.^11^, ^12^ These VWF “strings” provide scaffolds for the recruitment of circulating platelets and soluble plasma VWF, contributing to the formation of the primary hemostatic plug, but also potentially promoting microangiopathy.^2^, ^13^

The length of VWF strings generated upon exocytosis reflects the size of the WPBs in which VWF was stored. WPBs are cigar‐shaped organelles, whose length ranges ten‐fold, between 0.5 and 5.0 µm. Their size depends on the structural status of the endothelial Golgi apparatus where they form, and experimental manipulations causing Golgi fragmentation consistently result in the formation of only short WPBs.^14^ Short WPBs also form when VWF biosynthesis by endothelial cells is reduced, or following statin treatment, via Golgi fragmentation‐independent and ‐dependent mechanisms, respectively.^14^, ^15^ Organelle size is also regulated by the metabolic status of endothelial cells, through an AMPK/GBF1‐mediated signaling pathway^16^ and, transcriptionally, by upregulation of Krüppel‐like factor 2 (KLF2).^17^

Importantly, in vitro experiments have revealed that reducing WPB size results in the shortening of the VWF strings they generate and in much‐reduced recruitment of platelets and soluble circulating VWF to the endothelial surface.^14^, ^15^ Conversely, endothelial cells respond to raised glucose levels (mimicking hyperglycemia) by producing longer WPBs, suggesting a link between long VWF strings and thrombotic manifestations in diabetes,^16^ which is often associated with high levels of plasma VWF and microangiopathy.^7^

WPB size is, therefore, plastic and responds to physiological cues and pharmacological treatments. Such findings suggest that drug‐mediated reduction of WPB size might provide alternative or coadjuvant therapeutic approaches to current clinical interventions in thrombotic pathologies in which dysregulated formation and/or prolonged persistence of VWF strings on vascular walls play a triggering role.

We designed a screen to identify drugs that reduce WPB size and thus can potentially reduce endothelial pro‐thrombotic capacity. Out of 1280 human licensed drugs we found 37 compounds fitting our criteria, with a variety of mechanisms of action, implying that a number of cellular pathways influence biogenesis of WPBs.

## 2 METHODS

### 2.1 Cells

Human umbilical vein endothelial cells (HUVECs) were obtained commercially from PromoCell or Lonza. Cells were from pooled donors of both sexes expanded in our lab and used at low passage (3‐4), within 15 population doublings since isolation from umbilical cord. Cells were maintained in HGM (HUVEC growth medium) of the following composition: M199 (Gibco, Life Technologies), 20% fetal bovine serum (Labtech), 30 µg/mL endothelial cell growth supplement from bovine neural tissue, and 10 U/mL heparin (both from Sigma‐Aldrich). Cells were cultured at 37°C, 5% CO_2_, in humidified incubators.

### 2.2 Reagents

The library compounds (1280 FDA‐approved drugs), from Prestwick Chemical, were stored at −80°C as 10 mmol/L stock solutions in dimethylsulfoxide (DMSO). For the initial screen, compounds were transferred to Echo Qualified 384‐Well Low Dead Volume Microplates using the Echo^®^ 520 acoustic dispenser (both from Labcyte). Concentration‐response tests were carried out to confirm the activity of the primary hits and determine their WPB‐shortening EC_50_ or lowest active concentration. In these experiments, each compound, starting at 10 µmol/L, was diluted in two‐fold steps across the eight wells of a 96‐well plate columns (concentration range 78 nmol/L‐10 µmol/L), using the Echo^®^ 520 acoustic dispenser as described above. Each compound was tested in duplicate plates for independent measurements. Antibodies used in this study were: rabbit polyclonal anti‐VWF pro‐peptide region,^18^ kindly provided by Dr Carter (St. George's University, London); mouse monoclonal anti‐GM130 (clone 35) from BD Biosciences; a rabbit polyclonal anti‐VWF from DAKO (cat. no. A0082). DMSO (Hybri‐Max™, cat. No. D2650), nocodazole (cat. No. M1404), proscillaridin A (cat. No. PHL82774), cyclosporine A (cat. No. 30024) clomipramine (cat. No. C7291), and astemizole (cat No. A2861) were from Sigma‐Aldrich (now Merck Millipore). siRNA sequences targeting Luciferase and VWF were custom synthesized and previously described.^15^ Normal pooled human plasma (Cryochek™; cat. No. CCN‐15) was from Precision Biologic.

### 2.3 Drug screen

Human umbilical vein endothelial cells were seeded on gelatin‐coated 96‐well plates (Nunclon surface©, NUNC) at 15 000 cells/well and cultured. After 24 hours, cells were rinsed with fresh medium and then library compounds were added with the Echo^®^ 520 (Labcyte) acoustic dispenser and medium volume per well was adjusted with a MultiFlo FX dispenser (BioTek Instruments Inc) to 100 µL for a final compound concentration of 10 µmol/L. Plates were prepared in duplicate. Each plate contained one column treated with negative DMSO control (0.1%, final concentration) and one column treated with nocodazole positive control (3.3 µmol/L, final concentration). All cells (DMSO‐, nocodazole‐, and compound‐treated) received the same amount of DMSO vehicle (0.1% vol:vol). After 24 hours treatment, cells were rinsed twice with warm, fresh HGM and fixed by incubation with 4% formaldehyde in phosphate‐buffered saline (PBS; 10 minutes, RT).

### 2.4 Concentration‐response experiments

Human umbilical vein endothelial cells were seeded in 96‐well plates and pre‐processed as described for the screen. Hit compounds from the primary screen were obtained by the Prestwick library and delivered to cells using the Echo^®^ 520 acoustic dispenser (Labcyte) and cells were treated as described above. The concentrations of each compound ranged from 78 nmol/L to 10 µmol/L in two‐fold increments across the wells of one plate column. Duplicate plates were prepared. Each plate contained DMSO and nocodozale controls as detailed for the screen. Treatment was for 24 hours, at the end of which cells were fixed as described for the screen. Compounds were considered WPB‐shortening drugs if, at least at the highest concentration tested (10 µmol/L), their fractional area (FA) of long WPBs was ≤0.35. To determine the proscillaridin A EC_50_, a dedicated concentration‐response experiment was carried out with two‐fold dilutions along two columns of 96‐well plates in duplicate (concentration range 76 pmol/L‐2.5 µmol/L).

### 2.5 Immunostaining and image acquisition

Fixed cells were processed for immunostaining as previously described.^15^ WPBs were labelled using a rabbit polyclonal antibody to the VWF pro‐peptide region. The Golgi apparatus was labeled with an anti‐GM130 mAb. Primary antibodies were detected with Alexa Fluor dye‐conjugated antibodies (Life Technologies). Nuclei were counterstained with 33342 (Life Technologies). Images (nine fields of view per well) were acquired with an Opera High Content Screening System (Perkin Elmer) using a 40× air objective (NA 0.6). For small‐scale high‐throughput morphometry (HTM) experiments, imaging was carried out with similar parameters using either the Opera or the Opera Phenix High Content Screening Systems (Perkin Elmer).

### 2.6 High‐throughput morphometry (HTM) workflow

Image processing and extraction of morphological parameters (HTM) have been described in detail elsewhere.^14^ *R* scripts for image for data processing are available upon request. WPB size was expressed per well (ie, summing the values measured in the nine fields of view acquired per well) as the fraction of the total WPB area covered by WPBs > 2 µm. This morphometric approach was used also to measure WPB numbers and cell numbers (nuclei). WPB size was expressed per well (ie, summing the values measured in the nine fields of view acquired per well) as the fraction of the total WPB area covered by WPBs > 2 µm. This morphometric approach was used also to measure WPB numbers and cell numbers (nuclei). Normalization and scoring were done using the cellHTS2 (version 2.46.1) Bioconductor *R* package.^19^ DMSO negative controls were used for per plate median percent of control (POC) normalization. The *k*th compound value *x* in *i*th plate and replicate $x_{\mathrm{ki}}$ is relative to the average of within‐plate negative controls.

$$x_{\mathrm{ki}}^{\mathrm{POC}}=\frac{x_{\mathrm{ki}}}{\mu_{i}^{\mathrm{neg}}}\times 100$$

where $\mu_{i}^{\mathrm{neg}}$ is the average of the measurements on the negative controls in the *i*th plate and replicate. Normalized values were scored using robust z‐score method by subtracting the overall median from each measurement and dividing the result by the overall median absolute deviation. *R* scripts for data processing are available upon request. Concentration‐response data analysis was done in *R* language, using the DRC package by Christian Ritz and Jens C. Strebig (https://CRAN.R‐project.org/package=drc). The drm() function was used to fit a dose‐response model, a four‐parameter log‐logistic function (LL.4), applied to each dataset; four parameter values were calculated: slope, lower limit, upper limit, and EC_50_ value.

### 2.7 VWF and protein assays

For protein and VWF determinations, cells were cultured in 12‐well plates and subjected to drug treatments as described for HTM experiments. Cells were then rinsed twice with PBS and lysed with ice‐cold cold RIPA buffer (100 mmol/L Tris‐HCl, pH 7.5, 150 mmol/L NaCl, 1% TX‐100, 0.5% Na‐deoxycholate and 0.05% SDS) supplemented with protease inhibitor cocktail (P8340, Sigma‐Aldrich) and clarified by centrifugation. VWF cell content was measured by a sandwich enzyme‐linked immunosorbent assay (ELISA) as previously detailed.^15^ VWF amounts were calculated by converting the volumes of the pooled plasma used for standard curves into nanograms of VWF (1 µL human pooled plasma = 10 ng VWF). Protein content in HUVEC lysates was measured using the Pierce™ BCA Protein Assay Kit (Cat. No. 23225). Two biological replicates, each with three technical replicates, were analyzed in these experiments.

### 2.8 Plasma VWF recruitment assays

Human umbilical vein endothelial cells were seeded in HGM on gelatin‐coated Ibidi VI µ‐slides (Thistle Scientific). At 24 hours, cells were rinsed with three changes of fresh medium and drug treatment was started. After 24 hours, µ‐slides were attached to a pump system (Harvard Apparatus), while kept in a heated chamber (37°C) for the duration of the assay. Cells were initially perfused with Ca^2+^/Mg^2+^ Hanks’ balanced salt solution (HBBS; Life Technologies, Cat no. 14025092) supplemented with 0.2% bovine serum albumin (BSA) for 2 minutes. Cells were then perfused with HBBS containing 100 µmol/L histamine for a further 3 minutes in order to stimulate VWF exocytosis, followed by perfusion for 1 minute with human pooled plasma supplemented with histamine (Cryochek™; Precision Biologic, cat. no. CCN‐15). A constant flow rate was kept throughout the perfusion and produced a wall shear stress of 2.5 dynes/cm^2^ (0.25 MPa). Finally, cells were fixed by perfusion of 4% formaldehyde in PBS: 2 minutes at 2.5 dynes/cm^2^, followed 2 minutes at 1.25 dynes/cm^2^. Flow was then stopped and cells kept in fixative solution in static condition for 6 more minutes. Fixed cells were then rinsed with PBS. After blocking treatment with 5% (BSA) in PBS for 30 minutes, non‐permeabilized samples were incubated with a polyclonal anti‐VWF antibody (DAKO, cat. No. A00A2; 1:1000 in 1% BSA/PBS) for 1 hour, in order to label cell surface‐associated VWF. Samples were then incubated with an anti‐rabbit Alexa Fluor564‐conjugated antibody and Hoechst 33342 (both from Life Technologies) at 1:500 and 1:10 000, respectively, in 1% BSA/PBS for 45 minutes. Images were acquired at a spinning disc confocal microscope (UltraView VoX, Perkin Elmer) with a 20× objective. For each treatment, a tiled image panel (36 fields of view in a 3 × 12 grid) with a number of z‐slices sufficient to visualize the entire depth of the sample was acquired. Quantification of surface VWF was done with Fiji (ImageJ). Each field of view was assessed independently. Z‐stacks were converted into maximum intensity projections. VWF fluorescence signals were threshholded and converted into binary values. For each field of view, the area covered by the VWF signal was measured using ImageJ’s “Analyze particle” function. Experiments involving VWF level reduction by siRNA and statin treatment were carried out with as previously described.^15^ Each treatment was replicated with similar results in at least two independent experiments.

## 3 RESULTS

### 3.1 Screen

**FIGURE 1:**
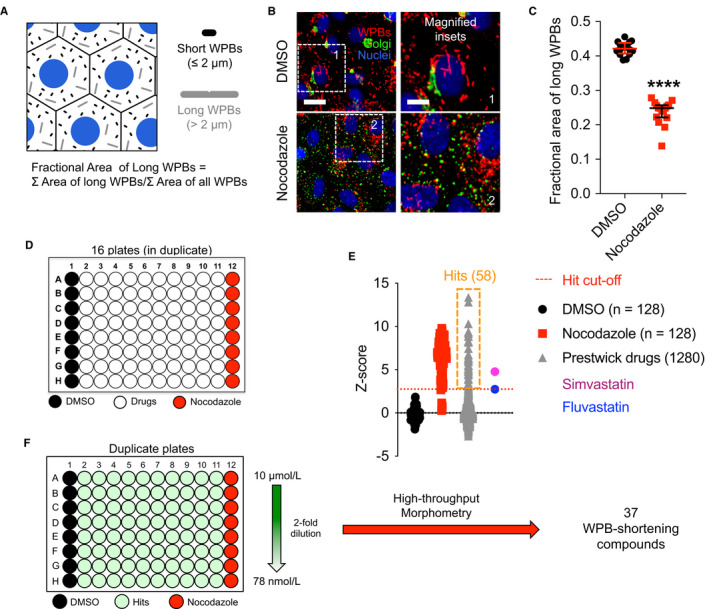
Drug screen: design, execution, and results. A, Weibel‐Palade body (WPB) sizes range between 0.5 and 5 µm. To measure a single quantitative parameter accounting for WPB size in an organelle population, we calculated the fraction of the area (FA) covered by long (ie, >2 µm) WPBs. B, Dimethylsulfoxide (DMSO) and nocodazole were used as negative and positive control treatments, respectively, for reducing WPB size. Micrographs show the effects of the two control treatments on human umbilical vein endothelial cells (HUVECs; 24 hours); scale bar: 20 µm (inset, 10 µm). C, Quantification of the “FA of long WPBs” for cells treated as in (B). Data‐points represent the values calculated for each well of a 96‐well plate; median and interquartile ranges are shown. ****, *P* < .0001; Mann‐Whitney test. D, Screen set‐up. Individual library drugs were dispensed into single wells of 96‐well plates, which also had two columns treated with DMSO and nocodazole controls. Two plates with identical drug layout (biological duplicates) were analyzed. E, Screen results. Z‐scores of the library drugs are plotted. Hit drugs were selected based on the effects of fluvastatin and simvastatin, which we previously showed to reduce WPB size. Fluvastatin, with the lowest Z‐score, was used as cut‐off for the selection of hit compounds. F, Concentration‐response experiments were carried out on the initial screen hits, by two‐fold dilutions (starting at 10 µmol/L) along 96‐well plate columns with each compound tested in duplicate plates. FA of long WPBs was measured for each drug concentration allowing calculation of their EC_50_ or identification of their lowest effective concentration in the range tested

A quantitative high‐throughput microscopy‐based workflow, HTM, allows rapid quantification of the size of tens to hundreds of thousands of WPBs within thousands of endothelial cells.^14^ HTM has been applied for analytical purposes and in phenotypic screens.^14^, ^15^, ^16^, ^20^, ^21^, ^22^ In the present report, HTM was deployed to identify compounds that can induce a reduction of WPB size in HUVECs. In cultured HUVECs, WPBs longer than 2 µm account for approximately 20% of these organelles and roughly 40% of the VWF they store.^14^ Thus, while a minority, these long WPBs are disproportionally important with respect to secreted endothelial VWF. For the purpose of the screen, we quantified WPB size as the ratio between the area covered by WPBs longer than 2 µm and the area covered by all these organelles; we define this parameter as “fractional area (FA) of long WPBs” (Figure 1A).^15^ The effect of each compound in the library on WPB size was compared to two controls: treatment with DMSO and nocodazole, the negative and positive controls, respectively, for WPB shortening^14^, ^15^ (Figure 1B, C). HUVECs were incubated with the 1280 compounds of the Prestwick library at 10 µmol/L for 24 hours in single wells of 96‐well plates, in duplicate (two separate plates; Figure 1D). After fixation, immuno‐staining, and image acquisition, the FA of long WPBs in treated cells was quantified. Per‐plate and inter‐plate normalization to DMSO controls was implemented in order to rank the effects of the library compounds on WPB size by Z‐score (Figure 1E; Table S1 in supporting information).

### 3.2 Hit selection and confirmation

Statins are cholesterol‐lowering drugs that inhibit the enzyme 3‐hydroxy, 3‐methylglutaryl CoA reductase (HMGCR) in the mevalonate pathway, upstream of the biosynthesis of cholesterol. We have previously shown that treatment of endothelial cells with two statins, simvastatin and fluvastatin, induces WPB size shortening, resulting in reduced adhesive properties of the VWF released by activated endothelial cells (HUVEC), measured by the reduced size of platelet‐decorated VWF strings and by the recruitment of VWF from a flowing plasma pool.^15^ Fluvastatin and simvastatin are present in the Prestwick library. We therefore used their Z‐score to establish a stringent cut‐off for selection of positive hits. This approach identified 58 compounds, 4.5% of the library (Figure 1E, orange box, simvastatin and fluvastatin).

Concentration‐response experiments were then carried out in order to both confirm compound activity and establish their EC_50_ or minimal effective concentrations. HUVECs were incubated for 24 hours with the 58 hits at concentrations ranging from 10 µmol/L to 78 nmol/L (Figure 1F). Following image analysis, if the fractional area of long WPBs was ≤0.35, at least at 10 µmol/L, compounds were confirmed as WPB‐shortening. This secondary screen confirmed that 37 licenced compounds (2.9% of the library) are capable of inducing WPB size reduction in 24 hours, with varied potency (Table 1). Of note, aside from simvastatin and fluvastatin, the other three statins present in the Prestwick library were confirmed as WPB‐shortening drugs (Table 1), indicating that both our screening approach and the criteria for hit selection were robust.

**Table 1:** WPB‐shortening compounds

Compound	EC_50_ ± SE or lowest active concentrations (µmol/L)	Compound	EC_50_ ± SE or lowest active concentrations (µmol/L)
Monensin (1)	≤78 nmol/L	Itraconazole (9, 18)	1.891 ± 0.407
Colchicine (2, 18)	≤78 nmol/L	Digoxigenin (3, 5)	2.172 ± 0.804
Podophyllotoxin (2)	≤78 nmol/L	Hexachlorophene (10)	2.376 ± 0.439
Proscillaridin A (3, 5)	0.018 ± 0.003	Mitoxantrone (6)	2.958 ± 0.356
Fluvastatin (4, 11)	0.198 ± 0.070	Parbendazole (2)	4.402 ± 0.986
Digoxin (3, 5, 18)	0.254 ± 0.047	Vorinostat (11)	4.622 ± 0.867
Lanatoside C (3, 5)	0.515 ± 0.203	Clomipramine (12, 13, 18)	4.699 ± 1.191
Cycloheximide (5)	0.532 ± 0.104	Verteporfin^a^	5 µmol/L
Simvastatin (4, 11, 18)	0.557 ± 0.195	Tegaserod (13)	10 µmol/L
Daunorubicin (6, 18)	0.662 ± 0.139	Bepridil (3, 14, 18)	10 µmol/L
Doxorubicin (6, 18)	0.678 ± 0.246	Maprotiline (12, 15)	10 µmol/L
Digitoxigenin (3, 5)	0.839 ± 0.062	Camptothecine (6)	10 µmol/L
Atorvastatin (4, 11, 18)	0.951 ± 0.312	Clomiphene (10)	10 µmol/L
Nocodazole (2)	1.046 ± 0.266	Astemizole (15, 18)	10 µmol/L
Cyclosporin A (7, 18)	1.071 ± 0.186	Thonzonium (1)	10 µmol/L
Aprepitant (8)	1.230 ± 0.153	Alclometasone (16)	10 µmol/L
Pyrvinium^a^	1.256 ± 0.143	Quinacrine (17, 18)	10 µmol/L
Lovastatin (4, 11, 18)	1.441 ± 0.261	Ciclesonide (16)	10 µmol/L
Mevastatin (4)	1.768 ± 0.213		

Note

WPB‐shortening compounds identified and confirmed in concentration‐response experiments in the range 0.078‐10 µmol/L. The EC_50_ (±standard error) or the lowest concentrations showing WPB‐shortening activity for each compound, within this concentration range, are indicated. For proscillaridin A, a cardiac glycoside, a second set of experiments was carried out to determine its EC_50_. The pharmacology (primary and other known targets) of each compound is indicated by the numbers within parentheses: 1, trans‐membrane pH gradient depleting agents; 2, microtubule polymerization inhibitors; 3, Na/K‐ATPase inhibitors; 4, HMGCR inhibitors/statins; 5, protein synthesis inhibitors; 6, DNA interaclating agents/topoisomerase inhibitors; 7, protein phosphatase inhibitors; 8, neurokinin receptor antagonists; 9, ergosterol synthesis inhibitors; 10, estrogen receptor agonists; 11, HDAC inhibitors; 12, noradrenaline reuptake inhibitors; 13, serotonin receptor antagonists and serotonin reuptake inhibitors; 14, voltage‐dependent Ca2 + inhibitors; 15, histamine receptor antagonists; 16, plucocorticord receptor agonist; 17, PLA2 inhibitors; 18, MDR1/ABCB1 inhibitors.

^a^ Pyrvinium and verteporfin have no known molecular targets.

### 3.3 Compound pharmacology

**FIGURE 2:**
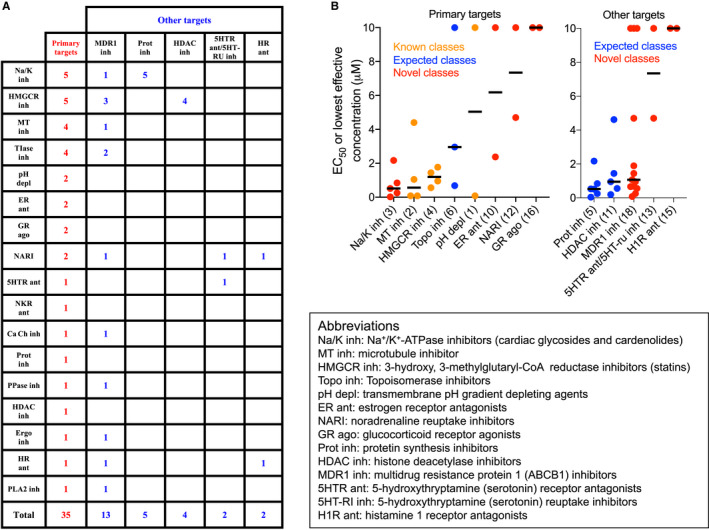
Classification of Weibel‐Palade body (WPB)‐shortening drugs. A survey of the mechanisms of action (see Table S2) identified molecular targets for 35 of the 37 compounds with WPB‐shortening activity. A, Based on the information retrieved, targets were classified as “primary” or “other” and represented in a tabular form in order to show the “cross‐talk” between pharmacological classes. B, Pharmacological classes with at least two compounds were graphically represented and ranked by “potency” using the median value (black bars) of their EC_50_ or lowest active concentration (the data points in the plots) identified in this work. The lowest value (78 nmol/L) was considered for those drugs found active at all concentrations tested in this work except for proscillaridin A, whose EC_50_ (18 nmol/L) was measured in a dedicated set of experiments. Numbering in parentheses is as in Table 1

A survey of the mechanisms of action of the 37 compounds showed that they had diverse pharmacology, consistent with the existence of a variety of mechanisms of WPB size control (Table S2 in supporting information). Based on their primary target or the biological activity affected, 26 drugs could be allocated into eight pharmacological classes, each with at least two compounds (Tables 1 and S2; Figure 2). Considering other known molecular targets of all the compounds and the cellular processes they that affect, five additional classes were identified. Several compounds were shared among pharmacological classes (Tables 1 and S2; Figure 2A).

Among the pharmacological classes inducing WPB shortening, we found microtubule (MT) depolymerizing agents; histone deacetylase (HDAC) inhibitors; topoisomerase inhibitors; and, as mentioned earlier, statins (HMGCR inhibitors). Compounds with these mechanisms of action have been shown to induce unlinking of the ribbon architecture of the Golgi apparatus, ie Golgi "fragmentation."^15^, ^23^, ^24^, ^25^ Because an intact Golgi ribbon is required for the biogenesis of long WPBs^14^ (see, for instance, nocodazole in Figure 1B), identification of these classes of molecules in our screen was expected. Previous work from our lab also showed that neutralization of the acidic lumen of WPBs disrupts the tubular structure of VWF, shifting the organelle shape from cylindrical to spherical, which is detected as shortening;^26^ and indeed we identified transmembrane pH gradient depleting agents. Reduction in VWF biosynthesis results in shorter WPBs without affecting the Golgi architecture^14^ and one of the screen hits, cycloheximide, is a classic protein synthesis inhibitor, while other compounds identified here have also been reported to have this activity (Figure 2B).^27^

Aside from those expected examples, entirely novel WPB‐shortening drug classes were also identified, including estrogen, glucocorticoid, serotonin and histamine receptor agonists or antagonists, noradrenaline reuptake inhibitors, and cardiac glycosides. Many of the identified compounds are shared by two or more pharmacological classes (Figure 2A), which may signal that these compounds mediate their effects on WPB size through yet unidentified common cellular processes and signaling pathways. In this respect, it is interesting that several compounds are also substrates and inhibitors of multidrug resistance protein 1, MDR1/ABCB1 (Tables 1 and S2; Figure 2A), an activity that may therefore hint at one of the cellular pathways that regulate WPB size.

**FIGURE 3:**
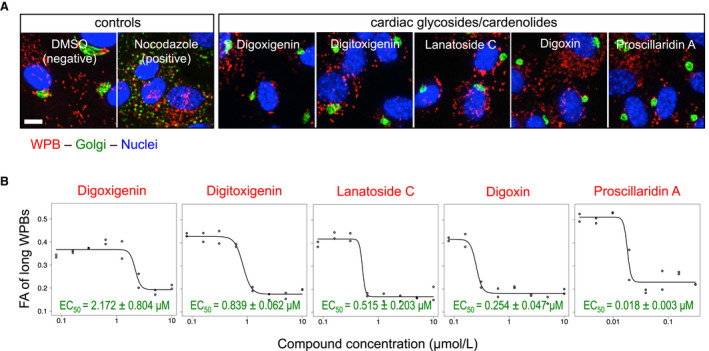
Cardiac glycosides and cardenolides. A, Original micrographs from the screen of human umbilical vein endothelial cells treated for 24 hours with 10 µmol/L each of the indicated compounds. Treatment with these molecules induces Weibel‐Palade body (WPB)‐shortening and Golgi apparatus compaction (compare to dimethylsulfoxide negative control). Scale bar: 10 µm. B, Concentration‐response curves of cardiac glycoside and cardenolide activity on WPB size; calculated EC_50_ values are reported

The cardiac glycosides and their cardenolide precursors were prominent among these novel pharmacological classes. These molecules, which inhibit Na^+^/K^+^‐ATPase (Tables 1 and S2), have a long clinical history in the treatment of congestive heart failure and atrial fibrillation and have recently attracted interest as potential anticancer molecules^28^ and senolytics, ie, selective inducers of senescent cell death.^29^ As a class, cardiac glycosides and cardenolides display the strongest WPB‐shortening activity (Figure 2B) and induce Golgi apparatus compaction (Figure 3A) instead of its fragmentation, suggestive of a novel WPB size‐reducing mechanism. In most cases, submicromolar concentrations of these compounds were sufficient to reduce WPB size, with proscillaridin A being effective at low nanomolar concentrations (Figure 3B; Table 1).

### 3.4 Hit validation

**FIGURE 4:**
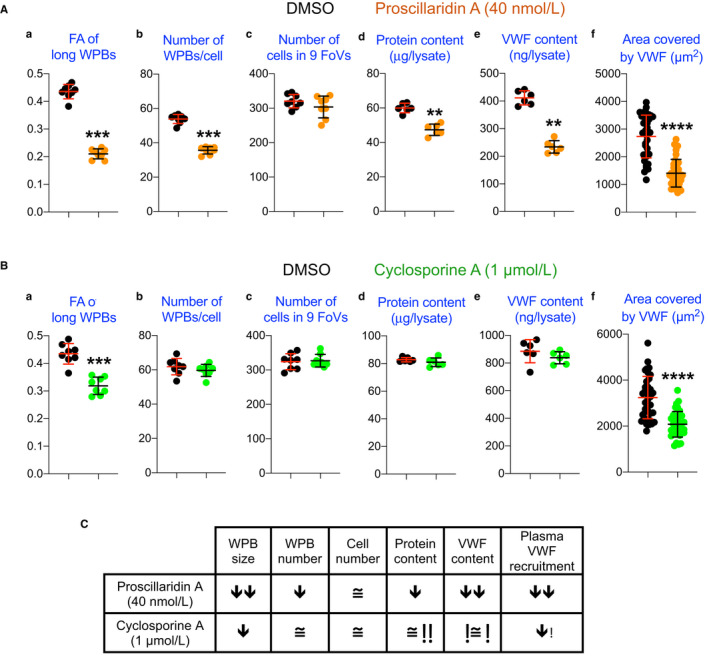
Antithrombotic effect of novel Weibel‐Palade body (WPB)‐size reducing compounds. WPB size and numbers, cell numbers, protein, and von Willebrand factor (VWF) content (a–e) of human umbilical vein endothelial cells treated with the cardiac glycoside proscilladirin (A) and the protein phosphatase inhibitor cyclosporine (B) at the concentration tested in VWF plasma recruitment assays (f). Data points in (a), (b), and (c) panels are from experiments done in 96‐well plates, with one column per treatment. Data points in panels (d) and (e) are samples in 12‐well plates (one data point per well). Data points in panels (f) are from 36 fields of view per treatment. Individual data points with median and interquartile ranges are shown. **, ***, and ****: *P* < .01, .001, and .0001, respectively (Mann‐Whitney test). C, Tabular summary of the experiments in A and B

We have shown that VWF exocytosis results in homotypic recruitment of the soluble VWF pool present in plasma to the stimulated endothelial surface and that treatments which reduce WPB size inhibit this process.^15^ We therefore tested whether novel compounds identified in our screen had this same effect. Cardiac glycosides and their aglycone precursors display a steep concentration/activity curve, meaning that their WPB‐shortening activity remains maximal down to and immediately disappears below their EC_50_ (Figure 3B). We therefore tested the effects of the most active of these compounds, proscillaridin A, at 40 nmol/L, a concentration ~ two‐fold higher than its EC_50_ (Figure 3B). Treatment of HUVECs with proscillaridin at this concentration resulted in strong WPB‐shortening, whose exocytosis generated, when compared to controls, reduced recruitment of plasma VWF (Figure 4A‐a, f). Because cardiac glycosides have been reported to inhibit protein synthesis,^27^ we assessed whether this might explain, through reduced VWF translation, their effect on WPB size. Protein and VWF levels in proscillaridin‐treated cells were reduced when compared to DMSO controls, although to different extents. VWF reduction (by ~50%), which was reflected not only in reduced WPB size but also by a fall in their numbers, was greater than the drop (by ~30%) in protein content (Figure 4A‐b, d, e). At 40 nmol/L, proscillaridin did not affect cell numbers (Figure 4A‐c), likely because in our experiments drug incubation started at 24 hours after seeding, allowing cells to become confluent and stop dividing. Interestingly, this finding indicates that this concentration of proscillaridin is not toxic to cells, in contrast to what we find with other drugs identified in our screen when tested at high concentration (Figure S1A, B in supporting information).

In a similar set of experiments, we also tested another novel WPB size‐reducing compound, cyclosporine A, an inhibitor of the protein phosphatase calcineurin, clinically used as an anti‐rejection drug in organ transplantation (Table S1).^30^ In contrast to cardiac glycosides, cyclosporine displays a more graded concentration‐dependent effect on WPB size (Figure S1C). We have documented that statin treatment at concentrations exerting submaximal effects on WPB size nevertheless result in decreased adhesive VWF activity.^15^ To verify whether this is a general feature of WPB size reduction, we tested cyclosporine at 1 µmol/L, approximately its EC_50_ (Table 1; Figure S1). At this concentration, cyclosporine induced a moderate reduction in WPB size (Figure 4B‐a); nevertheless, this size reduction was still efficient in diminishing soluble plasma VWF recruitment to the endothelial surface (Figure 4B‐f). Cyclosporine had no effect on the number of cells, total protein, or VWF levels (Figure 4B‐d, e).

## 4 DISCUSSION

Upon exocytosis, endothelial UL‐VWF self‐assembles into strings, which serve as recruiting platforms for platelets and circulating VWF, thus promoting the formation of the primary hemostatic plug.^15^, ^31^, ^32^ VWF strings also mediate pathological processes such as tumor metastasis, endocarditis, and microangiopathies.^13^, ^33^, ^34^ Interventions reducing the persistence and/or activity of VWF strings are therefore of interest as potential anti‐thrombotic therapies.

Modulation of organelle size has been suggested as a potential strategy to regulate biological functions and correct pathological states.^35^ In this perspective, WPBs represent a paradigmatic example. Reduction of WPB size has no effect on UL‐VWF formation, but blunts generation of long platelet‐decorated VWF strings following exocytosis and diminishes recruitment of plasma VWF to the endothelial surface.^14^, ^15^ Interventions that shorten WPBs could therefore provide alternative or coadjuvant therapies to current clinical interventions in thrombotic pathologies in which dysregulated formation and/or prolonged persistence of VWF strings play a triggering role.

In addition to the pharmacological and experimental manipulations disrupting the integrity of the Golgi apparatus,^14^, ^15^ formation of short WPBs is mediated by endogenous signaling pathways. We have uncovered a pathway involving AMPK‐dependent regulation of the Arf‐GEF GBF1, which is independent of alterations in the structure of the Golgi apparatus and links WPB size to the metabolic status of endothelial cells.^16^ Small WPBs are also generated upon overexpression of the transcription factor KLF2.^17^ KLF2 expression is promoted by athero‐protective flow patterns and induces transcriptional changes in hundreds of endothelial genes, resulting in anti‐inflammatory and antithrombotic cellular adaptations.^36^, ^37^, ^38^, ^39^ Treatment with statins also upregulates KLF2 expression; and their anti‐inflammatory, anticoagulant, and antithrombotic effects are believed to be mediated by this transcription factor.^40^, ^41^ However, WPB size reduction induced by statin treatment does not require KLF2.^15^ Altogether, a significant body of experimental evidence indicates that the size of WPBs is subject to regulation and represents a target for pharmacological intervention in hemostatic function and thrombotic risk.

We therefore screened licenced drugs with the aim of identifying WPB size‐reducing molecules that could be rapidly repurposed as antithrombotics. We found 37 drugs with this activity, the majority of which can be grouped into pharmacological classes (Table 1; Figure 2). Some of these classes, such as microtubule depolymerizing agents and statins, have been identified by previous work.^14^, ^15^ Others might be expected, due to their effects on the Golgi ribbon, as in the case of HDAC and topoisomerase inhibitors.^24^, ^25^ Our screen also identified compounds with pharmacology previously unknown to affect WPB biogenesis and size. Together, these findings suggest that several cellular pathways can modulate the size of WPBs produced by endothelial cells.

MDR1 is an organic anion transporter. Its upregulation is responsible for the development of tumor resistance to chemotherapy, hence its name. While this activity toward xenobiotics was the first to be identified, it has become clear that MRP1 is also involved in the cellular efflux of endogenous molecules, mediating pro‐inflammatory signaling pathways and may act as an oxidative stress sensor.^42^ Interestingly, more than a third of the novel WPB‐shortening compounds with varied mechanisms of action (Table 1; Figure 2; Table S2) have also been described as MDR1 substrates and inhibitors. This suggests the possibility that, in addition to their main molecular target, these drugs could affect the efflux of endogenous MDR1 signaling substrates, which regulate WPB size through a mechanism to be elucidated by future investigations.

Because the screen endpoint was 24 hours, the drugs we identified are relatively fast‐acting. Several of the compounds display activity between submicromolar and low nanomolar concentrations (Table 1), which is likely to be compatible with their use in the clinic. The present study confirms statins’ effects on WPB size remodeling.^15^ Statins are known to rapidly produce anti‐inflammatory and anticoagulant effects on the endothelium^43^ and their acute administration in the context of percutaneous angioplasty greatly reduces postoperative myocardial infarctions.^44^ The compounding of these fast‐acting effects, WPB size reduction included, suggests that statins may represent a promising tool for acute, emergency treatment in endotheliopathies involving inflammation, coagulation, and thrombosis.

As a class, the most potent WPB‐shortening drugs identified in this study were the cardiac glycosides and their cardenolide precursors (Figure 2B; Table 1). Our testing of proscillaridin A, the most active among these compounds, shows that its WPB‐shortening activity results in reduced plasma VWF recruitment to the endothelial surface, an in vitro antithrombotic phenotype (Figure 4A). It is worth noting that the proscillaridin concentration used in these assays is comparable to its plasma concentration in patients.^45^

### 4.1 Considerations on drug toxicity

Our findings indicate that, with the caution due to their known dose‐dependent cytotoxicity,^46^ cardiac glycosides may be worth exploring in acute and, perhaps, chronic antithrombotic therapies. Another novel WPB‐shortening compound, cyclosporine A, was tested at a submaximal concentration with respect to its WPB‐shortening activity. Despite the moderate effects on WPB size, cyclosporine at 1 µmol/L caused a reduction in plasma VWF recruitment to the endothelial surface in our in vitro antithrombotic assay (Figure 4B‐f). As for proscillaridin, it is worth pointing out that this concentration is comparable to those that cyclosporine reaches in patient plasma after administration.^47^ Proscillaridin and cyclosporine seem to act on WPB size through different mechanisms (Figure 4C). Our preliminary characterization suggests that proscillaridin reduces global protein synthesis, confirming a previous study on cardiac glycosides.^27^ Interestingly, its effects on VWF levels are more pronounced than those on protein synthesis (Figure 4A‐d, e). This finding might indicate either some specificity of proscillaridin in reducing VWF synthesis or increasing its secretion or exerting an effect on its intracellular degradation, perhaps through WPB autophagy.^48^, ^49^, ^50^ We rule out changes in VWF secretion, because in cells treated with another cardiac glycoside, lanatoside C (at 10 µmol/L), while the cellular VWF levels were reduced, as a percentage of the total both its basal and histamine‐stimulated release were identical to those of DMSO‐treated controls (not shown), an indication that storage and secretion of VWF were not affected by this glycoside. Regardless of the specific mechanism, our data suggest that at the concentration tested in our functional assays, proscillaridin is not cytotoxic, given that after 24 hours’ incubation it did not reduce cell numbers, as opposed to two other compounds we tested at high concentration, clomipramine and astemizole (Figure 4A and S1). At the concentration tested, cyclosporine resulted in no changes of the parameters we measured except for a modest reduction in WPB size and its effects on plasma VWF adhesion (Figure 4B). Interestingly, calcineurin inhibition by cyclosporine results in activation of AMPK.^51^, ^52^ Because AMPK is a positive regulator of GBF1 activity, which in turn reduces WPB size,^16^ cyclosporine indirect activation of this kinase could explain its effects on WPB size. Cyclosporine was also reported to stimulate UL‐VWF secretion, which might explain the microangiopathic manifestations observed in a subset of organ transplant patients on anti‐rejection therapy.^53^ However, another study appears to contradict these conclusions, as specific inhibition of the calcineurin pool involved in WPB exocytosis results in decreased VWF secretion.^54^ While these reports suggest caution in the adoption of cyclosporine in antithrombotic therapies, our findings nevertheless warrant its investigation in this clinical context.

The compounds identified here are licensed and some have a decades‐long history of clinical use. Their toxicity and therapeutic profiles are therefore well known. On the back of this knowledge, clinicians could design protocols for the safe testing of the most active WPB‐shortening drugs in antithrombotic trials. In the context of potential drug toxicity, we would like to note that combination of WPB size reducing treatments, acting through different mechanisms, can display additive effects in the abatement of in vitro plasma VWF recruitment to the endothelial surface (Figure S1D). In a clinical setting, antithrombotic effects might therefore be achieved by administering combinations of drugs at submaximal concentrations with respect to their WPB‐shortening activity.

### 4.2 Hypercoagulability in COVID‐19

The disease caused by SARS‐CoV‐2 (COVID‐19) displays a range of symptoms, the most immediately apparent being respiratory. However, testing of hospitalized patients has uncovered clinical manifestations including hematological alterations that correlate with poor prognosis. Some clinicians have described the hypercoagulability observed in critically ill patients as a form of disseminated intravascular coagulation (DIC).^55^, ^56^, ^57^, ^58^, ^59^, ^60^, ^61^ However, other retrospective studies and case reports have shown that these patients have unusually high levels of plasma VWF, suggesting that the hypercoagulability observed may be dependent on intravascular thrombosis.^61^, ^62^, ^63^, ^64^

We hope that the drug dataset reported here will provide clinicians and researchers with licensed candidates that may potentially progress to clinical trials for the treatment of the thrombotic complications of COVID‐19. Apart from colchicine, which is being currently evaluated for COVID‐19 therapy (https://clinicaltrials.gov/ct2/show/NCT04322682), we are not aware of other ongoing clinical trials in which the compounds we have identified are being tested. Our findings, however, prompt us to suggest that in the event of such trials, it might be important to also evaluate the hematological effects of the compounds under consideration.

In conclusion, the present study confirms the hypothesis that reduction in WPB size correlates with the diminished adhesive activity of its VWF cargo. The identification of several licensed compounds with fast‐acting WPB‐shortening activity, two of which we prove effective at concentrations compatible with their use in the clinic, paves the way to their repurposing for novel acute antithrombotic therapies involving organelle size modulation.

## CONFLICTS OF INTEREST

The authors have no conflicts of interest to declare.

## AUTHOR CONTRIBUTIONS

FF, FP, RK, and DFC designed the study. FF, FP, and JRC did experiments. JK‐V, FP, and FF analyzed data. RK provided fundamental reagents and instrumentation. FF and DFC wrote the manuscript.

## Supporting information

Supplementary Material
