# Supplementary material for: Modulation of endothelial organelle size as an antithrombotic strategy
Source: J Thromb Haemost. 2020 Oct 16;18(12):3296–308. doi: 10.1111/jth.15084 (PMC8436738; doi:10.1111/jth.15084)
Supplement: Supplementary file 1 — Supplementary Material [file JTH-18-3296-s001.pdf]

Supporting information

Modulation of endothelial organelle size as an antithrombotic strategy

Francesco Ferraro[1,2,§*], Francesca Patella[1§], Joana R. Costa[3,4], Robin Ketteler[3], Janos Kriston-Vizi[5], Daniel F. Cutler[1*]

[1] Endothelial Cell Biology Group, MRC Laboratory for Molecular Cell Biology, University College London, Gower Street, London, WC1E 6BT

[2] Current address, Department of Biology and Evolution of Marine Organisms (BEOM), Stazione Zoologica Anton Dohrn, Villa Comunale, 80121 Naples

[3] Cell Signalling and Autophagy Group, MRC Laboratory for Molecular Cell Biology, University College London, Gower Street, London, WC1E 6BT

[4] Current address, Leukaemia Biology Research Group, Department of Haematology, Cancer Institute, University College London, 72 Huntley Street, London, WC1E 6DD

[5] Bioinformatics Image Core (BIONIC), MRC Laboratory for Molecular Cell Biology, University College London, Gower Street, London, WC1E 6BT

[§] These authors contributed equally

* Correspondence

Daniel F. Cutler
Phone: +44 (0)20 7679 7808
Email: d.cutler@ucl.ac.uk

Francesco Ferraro
Phone: +39 081 5833301
Email: francesco.ferraro@szn.it

Contains

Supplemental Table 1

Supplemental Table 2

Supplemental Figure 1

Supplemental Table 1. Prestwick library compounds identified as WPB-shortening in the primary screen. Z-scores for the compounds are shown.

Compound	Z-score	Compound	Z-score
Monensin	13.35	Vorinostat	5.4
Tegaserod	12.8	Clomiphene	5.17
Colchicine	10.3	Astemizole	5.14
Digoxin	9.39	Perhexiline	5.06
Aprepitant	9.33	Thonzonium	4.95
Lanatoside C	9.16	Parbendazole	4.91
Digoxigenin	8.87	Alclometasone	4.8
Bepridil	8.86	Pyrvinium	4.79
Proscillaridin A	8.83	Simvastatin	4.78
Chlormezanone	8.33	Imatinib	4.59
Digitoxigenin	8.3	Loperamide	4.48
Maprotiline	8.2	Dilazep	4.37
Podophyllotoxin	8.16	Daunorubicin	4
Nocodazole	7.45	Quinacrine	3.99
Mitoxantrone	7.44	Thioproperazine	3.95
GBR 12909	6.76	Fluspirilene	3.84
Hexachlorophene	6.69	Fluphenazine	3.44
Lovastatin	6.43	Ciclesonide	3.42
Clofazimine	6.39	Clomipramine	3.41
Camptothecine	6.3	Doxorubicin	3.37
Cycloheximide	6.3	Trifluoperazine	3.34
Ambroxol	6.2	Azithromycin	3.26
Adenosine 5'-monophosphate	6.13	Epirubicin	3.15
Methyldopa (L,-)	5.97	Carvedilol	3.05
Etoposide	5.9	Prochlorperazine	2.96
Cyclosporin A	5.55	Sertindole	2.95
Atorvastatin	5.52	Verteporfin	2.84
Mevastatin	5.47	Clemizole	2.77
Itraconazole	5.46	Fluvastatin	2.74

Supplemental Table 2. Pharmacology of WPB-shortening compounds.

**Compound**	**Known pharmacology**
Monensin	Ionophore antibiotic (Na+/H+ antiporter) produced by the fungus *Streptomyces cinnamonensis*. Depletes the transmembrane pH gradient of the Golgi apparatus and acidic organelles.<br>**PubMed:** https://pubmed.ncbi.nlm.nih.gov/2160275/<br>**PubMed:** https://pubmed.ncbi.nlm.nih.gov/23509771/
Tegaserod	Agonist of serotonin receptor 5HT4. Antagonist of 5HT2A, 5HT2B and 5HT2C serotonin receptors. Inhibitor of sodium-dependent serotonin transporter SLC6A4.<br>**DrugBank:** https://www.drugbank.ca/drugs/DB01079
Colchicine	Inhibitor of Tubulin beta-1 chain and Tubulin beta chain (TUBB1, TUBB) and of microtubule polymerization. Inhibits several CYP enzymes and transporters. Inhibits multidrug resistance protein 1 (MDR1/ABCB1).<br>**DrugBank:** https://www.drugbank.ca/drugs/DB01394
Digoxin	Cardiac Glycoside, consisting of three sugars linked to the aglycone digoxigenin. Inhibits Na/K ATPase alpha-1 (ATP1A1). Substrate, inhibitor and inducer of multidrug resistance protein 1 (MDR1/ABCB1). It was reported to inhibit protein synthesis. With digoxigenin and strophanthidin was shown to inhibit autosis (autophagic cell death) through Na^{+}/K^{+}-ATPase.<br>**PubMed:** https://pubmed.ncbi.nlm.nih.gov/20016840/.<br>**PubMed:** https://pubmed.ncbi.nlm.nih.gov/24277826/<br>**DrugBank:** https://www.drugbank.ca/drugs/DB00390
Aprepitant	Antiemetic; it is a substance P/Neurokinin 1 (NK1) receptor antagonist.<br>**DrugBank;** https://www.drugbank.ca/drugs/DB00673
Lanatoside C	Cardiac Glycoside. Inhibits Na/K ATPase alpha-1 (ATP1A1). Like other cardiac glycosides and cardenolides, it was reported to inhibit protein synthesis. Possible inhibitor of autosis (autophagic cell death) through Na^{+}/K^{+}-ATPase.<br>**PubMed:** https://pubmed.ncbi.nlm.nih.gov/20016840/<br>**PubMed:** https://pubmed.ncbi.nlm.nih.gov/24277826/<br>**KEGG drug database**: https://www.genome.jp/dbget-bin/www_bget?dr:D01972
Digoxigenin	Cardenolide precursor of digoxin. Inhibitor of Na/K-ATPase subunit alpha-1 (ATP1A1). Like other cardiac glycosides and cardenolides, it was reported to inhibit protein synthesis. Possible inhibitor of autosis (autophagic cell death) through Na^{+}/K^{+}-ATPase.<br>**PubMed:** https://pubmed.ncbi.nlm.nih.gov/20016840/<br>**PubMed:** https://pubmed.ncbi.nlm.nih.gov/20388710/<br>**PubMed:** https://pubmed.ncbi.nlm.nih.gov/24277826/<br>**DrugBank:** https://www.drugbank.ca/drugs/DB03671
Bepridil	Inhibitor of voltage-sensitive Ca2+ channels CACNA1A, CACNA1H, CACNA2D2. Inhibits Na/K-ATPase subunit alpha-1 (ATP1A1). Inhibits voltage-gated potassium channel KCNQ1. Inhibits calcium/calmodulin dependent 3',5'-cyclic nucleotide phosphodiesterases PDE1A and PDE1B. Inhibits multidrug resistance protein 1 (MDR1/ABCB1)<br>**DrugBank:** https://www.drugbank.ca/drugs/DB01244
Proscillaridin A	Cardiac Glycoside. Inhibits Na/K ATPase alpha-1 (ATP1A1). Like other cardiac glycosides and cardenolides, it was reported to inhibit protein synthesis. Possible inhibitor of autosis (autophagic cell death) through Na^{+}/K^{+}-ATPase.<br>**PubMed:** https://pubmed.ncbi.nlm.nih.gov/20016840/<br>**PubMed:** https://pubmed.ncbi.nlm.nih.gov/24277826/<br>**KEGG drug database**: https://www.kegg.jp/dbget-bin/www_bget?dr:D01379

Digitoxigenin	Cardenolide steroid precursor of the cardiac glycoside digitoxin. Inhibitor of Na/K-ATPase subunit alpha-1 (ATP1A1). Like other cardiac glycosides and cardenolides, it was reported to inhibit protein synthesis. With digoxin and strophanthidin was shown to inhibit autosis (autophagic cell death) through Na^{+}/K^{+}-ATPase.<br>**PubMed:** https://pubmed.ncbi.nlm.nih.gov/20016840/<br>**PubMed:** https://pubmed.ncbi.nlm.nih.gov/20388710/<br>**PubMed:** https://pubmed.ncbi.nlm.nih.gov/24277826/<br>**KEGG drug database:** https://www.kegg.jp/dbget-bin/www_bget?C08876
Maprotiline	Tetracyclic antidepressant with similar pharmacological properties to tricyclic antidepressants (TCAs). Similar to TCAs, it inhibits neuronal norepinephrine reuptake, possesses some anticholinergic activity, and does not affect monoamine oxidase activity. It differs from TCAs in that it does not appear to block serotonin reuptake. Inhibits Sodium-dependent noradrenaline transporter (SLC6A2). Antagonist of Histamine H1 receptor (HRH1), Muscarinic acetylcholine receptors M1, M2, M3 and M4, alpha 1 and 2 adrenergic receptors. Binds (no pharmacological action established) with Dopamine D2 receptor and serotonin receptors HTR2A, HTR2C. It is an antagonist of HTR7.<br>**DrugBank:** https://www.drugbank.ca/drugs/DB00934
Podophyllotoxin	A lignan found in podophyllin resin from the roots of podophyllum plants. It is a potent spindle poison. Inhibitor of DNA topoisomerase 2-alpha (TOP2A) and Tubulin alpha-4A chain and beta chain. KEGG reports it as an antineoplastic, antiviral and Tubulin polymerization inhibitor.<br>**DrugBank:** https://www.drugbank.ca/drugs/DB01179<br>**KEGG drug database:** https://www.kegg.jp/dbget-bin/www_bget?dr:D05529
Nocodazole	It is a benzimidazole, an organic compound containing a benzene ring fused to an imidazole ring. It binds Tubulin and depolymerizes microtubules. Described as antineoplastic in the KEGG drug database.<br>**PubMed:** https://pubmed.ncbi.nlm.nih.gov/343926/<br>**KEGG drug database:** https://www.kegg.jp/dbget-bin/www_bget?dr:D05197
Mitoxantrone	Inhibitor of DNA topoisomerase 2-alpha (TOP2A). DNA intercalating molecule.<br>**DrugBank:** https://www.drugbank.ca/drugs/DB01204
Hexachlorophene	Chlorinated bisphenol antiseptic with a bacteriostatic action against Gram-positive organisms, but much less effective against Gram-negative organisms. Inhibits E. coli D-lactate dehydrogenase. Inhibits human Succinate dehydrogenase and Glutamate dehydrogenase 1. Competitive antagonist of estrogen receptor (ESR1).<br>**DrugBank:** https://www.drugbank.ca/drugs/DB00756
Lovastatin	It is a statin; i.e., an inhibitor of hydroxymethylglutaryl coenzyme A (HMG-CoA) reductase inhibitor (HMGCR). Was shown to inhibit Histone deacetylases, like other statins and Multi-drug resistance protein 1 (MDR1/ABCB1)<br>**PubMed:** https://pubmed.ncbi.nlm.nih.gov/18381445/<br>**PubMed:** https://pubmed.ncbi.nlm.nih.gov/11474784/<br>**DrugBank:** https://www.drugbank.ca/drugs/DB00227
Camptothecine (S,+)	Alkaloid isolated from the stem wood of the Chinese tree *Camptotheca acuminata*. It selectively inhibits the nuclear enzyme DNA topoisomerase type I. Several semisynthetic analogues of camptothecine have demonstrated antitumor activity. Binds DNA and DNA topoisomerase 1 (TOP1) forming a stable ternary complex that prevents ligation activity of TOP1.<br>**DrugBank:** https://www.drugbank.ca/drugs/DB04690
Cycloheximide	Inhibitor of protein synthesis.<br>**PubMed:** https://pubmed.ncbi.nlm.nih.gov/20118940/

Cyclosporin A	Lipophilic cyclic polypeptide formed by 11 amino acids with immunosuppressive and immunomodulatory properties. Essentially, a calcineurin inhibitor, resulting in inhibition of T cell activation. It binds to the intracellular receptor cyclophilin-1 forming a cyclosporin-cyclophilin complex that inhibits calcineurin, which prevents the dephosphorylation and activation of the nuclear factor of activated T cells (NF-AT). The NF-AT is a transcription factor that regulates the production of pro-inflammatory cytokines such as IL-2, IL-4, interferon-gamma and TNF-alpha. Cyclosporin inhibits calcineurin regulatory subunit B type 2 (PPP3R2) and binds Peptidyl-prolyl cis-trans isomerase A, F (PPIA, PPIF) and calcium signal-modulating cyclophilin ligand CAMLG. Cyclosporyn A is a substrate, inhibitor and inducer of multidrug resistance protein 1 (MDR1/ABCB1).<br>**DrugBank;** https://www.drugbank.ca/drugs/DB00091
Atorvastatin	A statin; i.e., a hydroxymethylglutaryl coenzyme A reductase (HMGCR) inhibitor. Also inhibits dipeptidyl peptidase 4 (DPP4). Agonist of aryl hydrocarbon receptor (AHR). Inhibits multidrug resistance protein 1 (MDR1/ABCB1). It has been shown that other statins inhibit this transporter. Inhibits Solute carrier organic anion transporter family member 1A2, 1B1 (SLCO1A2, SLCO1B1). It is a substrate of several transporters. Like other statins, it inhibits histone deacetylases (HDAC) 1 and 2<br>**PubMed:** https://pubmed.ncbi.nlm.nih.gov/11474784/<br>**PubMed:** https://pubmed.ncbi.nlm.nih.gov/18381445/<br>**DrugBank:** https://www.drugbank.ca/drugs/DB01076
Mevastatin	A statin; it inhibits hydroxymethylglutaryl coenzyme A reductase (HMGCR). It is the first statin discovered and isolated from *Penicillium citinium*. It also inhibits liver carboxylesterase 1, CES1 (a triglyceride lipase).<br>Other statins inhibit histone de-acetylases. While possible that also mevastatin possesses this activity, this was not shown experimentally. Its activity on Multi-drug resistance protein 1 (MDR1/ABCB1) has not been tested.<br>**DrugBank:**https://www.drugbank.ca/drugs/DB6693
Itraconazole	A triazole antifungal agent. Inhibits cytochrome P450-dependent enzymes resulting in impaired ergosterol synthesis. It has been used against histoplasmosis, blastomycosis, cryptococcal meningitis and aspergillosis. Inhibits Lanosterol 14-alpha demethylase (CYP51A1) and several other CYP enzymes. Inhibits multidrug resistance proteins1 (MDR1/ABCB1) and SLCO2B1.<br>Among the triazole antifungal agents is the only one shown to inhibit the hedgehog pathway and angiogenesis.<br>**PubMed:** https://pubmed.ncbi.nlm.nih.gov/20385363/<br>**PubMed:** https://pubmed.ncbi.nlm.nih.gov/21896639/<br>**DrugBank:** https://www.drugbank.ca/drugs/DB01167
Vorinostat	Known also as suberoylanilide hydroxamic acid (SAHA), this compound is under investigation for the treatment of cutaneous T cell lymphoma (CTCL), a type of skin cancer. It is the first in a new class of agents known as histone de-acetylase inhibitors. Inhibits Histone deacetylase 1, 2, 3, 6 and 8 (HDAC1, HDAC2, HDAC3, HDAC6 and HDAC8). HDAC inhibitors and DNA-damaging (DNA-intercalating) agents were identified as novel Golgi disruptors.<br>**PubMed:** https://pubmed.ncbi.nlm.nih.gov/29074567/<br>**DrugBank:** https://www.drugbank.ca/drugs/DB02546
Clomiphene	Estrogen receptor (ESR1) agonist or antagonist, depending on the tissue. Inhibits some Cytochrome P450 (CYPs) enzymes.<br>**DrugBank:** https://www.drugbank.ca/drugs/DB00882

Astemizole	Long-acting, non-sedating, second generation antihistamine used in the treatment of allergy symptoms. Antagonist of Histamine H1 receptor (HRH1). Inhibitor of Potassium voltage-gated channel subfamily H member 2 (KCNH2). Also acts (with unclear pharmacology) on Potassium voltage-gated channel subfamily H member 1 (KCNH1) and Microtubule-associated protein tau (MAPT). Inhibits multidrug resistance protein 1 (MDR1/ABCB1)<br>**DrugBank:** https://www.drugbank.ca/drugs/DB00637
Thonzonium	Monocationic surface-active agent with surfactant and detergent properties. It is widely used to enhance dispersion and penetration of cellular debris and exudate, thereby promoting tissue contact of the administered medication. Inhibits V-ATPase, V-type proton ATPase subunit C 1 (ATP6V1C1).<br>**DrugBank:** https://www.drugbank.ca/drugs/DB09552
Parbendazole	Reported as an anthelmintic.<br>**KEGG drug database:** https://www.genome.jp/dbget-bin/www_bget?D05365<br>It also is known as a microtubule inhibitor.<br>**PubMed:** https://pubmed.ncbi.nlm.nih.gov/7031071/
Alclometasone	Synthetic glucocorticoid steroid for topical use in dermatology as anti-inflammatory, antipruritic, antiallergic, antiproliferative and vasoconstrictive agent. Agonist of Glucocorticoid receptor (NR3C1).<br>**DrugBank:** https://www.drugbank.ca/drugs/DB00240
Pyrvinium	Anthelmintic effective for pinworms; no pharmacological target is reported in DrugBank. Its pamoate salt displays preferential toxicity for various cancer cell lines during glucose starvation.<br>**PubMed:** https://pubmed.ncbi.nlm.nih.gov/15298733/<br>**DrugBank:** https://www.drugbank.ca/drugs/DB06816
Simvastatin	Inhibitor of 3-hydroxy-3-methylglutaryl-coenzyme A reductase (HMGCR). Affects (no defined mechanism) Integrin beta-2 (ITGB2) and inhibits Integrin alpha-L (ITGAL). Substrate of several CYP enzymes it also inhibits some of them. Inhibitor of Histone Deacetylases (HDAC) 1 and 2. Inhibitor of multidrug resistance protein 1 (MDR1/ABCB1).<br>**PubMed:** https://pubmed.ncbi.nlm.nih.gov/18381445/<br>**PubMed:** https://pubmed.ncbi.nlm.nih.gov/11474784/<br>**DrugBank:** https://www.drugbank.ca/drugs/DB00641
Daunorubicin	Very toxic anthracycline aminoglycoside antineoplastic isolated from *Streptomyces peucetius* and others yeasts. Used in treatment of leukaemia and other neoplasms. Targets DNA (DNA intercalating agent). Inhibits DNA topoisomerase 2-alpha (TOP2A) and DNA topoisomerase 2-beta (TOP2B). Substrate of CYP enzymes. Reported as substrate and inhibitor, but also inducer, of multidrug resistance protein 1 (MDR1/ABCB1). Inhibitor of multidrug resistance-associated protein 1 (ABCC1) and other ACB transporters.<br>**DrugBank**: https://www.drugbank.ca/drugs/DB00694
Quinacrine	Also known as mepacrine, is an acridine derivative formerly used as an antimalarial but superseded by chloroquine in recent years. Also used as an anthelmintic and in the treatment of giardiasis and malignant effusion and in cell biological experiments as an inhibitor of phospholipase A2. Targets DNA (DNA intercalating agent). Inhibits 85/88 kDa calcium-independent phospholipase A2 (PLA2G6), cytosolic phospholipase A2 (PLA2G4A), Inactive phospholipase C-like protein 1 (PLCL1), histamine N-methyltransferase (HNMT), multidrug resistance protein 1 (MDR1/ABCB1).<br>**DrugBank:** https://www.drugbank.ca/drugs/DB01103
Ciclesonide	Glucocorticoid used to treat obstructive airway diseases. Agonist of Glucocorticoid

	receptor (NR3C1).<br>**DrugBank:** https://www.drugbank.ca/drugs/DB01410
Clomipramine	3-chloro analogue of imipramine, a dibenzazepine-derivative tricyclic antidepressant (TCA). Inhibits Sodium-dependent serotonin transporter (SLC6A4), Sodium-dependent noradrenaline transporter (SLC6A2). Antagonist of 5-hydroxytryptamine receptor 2A (HTR2A), 5-hydroxytryptamine receptor 2B (HTR2B), 5-hydroxytryptamine receptor 2C (HTR2C). Inhibits Glutathione S-transferase P (GSTP1) and multidrug resistance protein 1 (MDR1/ABCB1).<br>**DrugBank:** https://www.drugbank.ca/drugs/DB01242
Doxorubicin	Cytotoxic anthracycline antibiotic isolated from cultures of *Streptomyces peucetius* var. *caesius*. Targets DNA (DNA intercalating agent). Inhibits DNA topoisomerase 2-alpha (TOP2A). Substrate of NOS 1,2 and 3. Substrate, inhibitor and inducer of multidrug resistance protein 1 (MDR1/ABCB1) and other ABC transporters.<br>**DrugBank:** https://www.drugbank.ca/drugs/DB00997
Verteporfin	A benzoporphyrin derivative, it is used as a photosensitizer for photodynamic therapy to eliminate the abnormal blood vessels in the eye associated with conditions such as the wet form of macular degeneration. No molecular targets reported.<br>**DrugBank:** https://www.drugbank.ca/drugs/DB00460
Fluvastatin	A statin, it inhibits 3-hydroxy-3-methylglutaryl-coenzyme A reductase (HMGCR). Like other statins has been reported to inhibit HDAC 1 and 2.<br>It is a substrate for several transporters.<br>**PubMed:** https://pubmed.ncbi.nlm.nih.gov/18381445/<br>**DrugBank:** https://www.drugbank.ca/drugs/DB01095

Figure S1. Additional data. A and B**.** In concentration-response experiments, clomipramine and astemizole were effective in shortening WPB size at 10 µmol/L (see Table 1, main text). To test whether higher concentrations of these compounds would induce a stronger effect, titration experiments were carried out. Both compounds did cause stronger organelle shortening at 20 and 50 µmol/L. However, these effects resulted from cytotoxicity, as shown by cell depletion from the wells. Clomipramine was toxic at concentrations > 20 µmol/L, while astemizole was toxic above 10 µmol/L. Experiments were carried out as detailed in the legend of Figure 4. *, ** and ***: $P < 0.05$, 0.01 and 0.001, respectively (Mann-Whitney test). C, concentration-response curve of Cyclosporine A. D, additive effects of the combination of two WPB-size reducing treatments. WPB size was reduced by decreasing cellular VWF level or by statin incubation or their combination. ****, $P < 0.0001$. Mann-Whitney test. Black asterisks label comparisons with Luciferase-siRNA/DMSO control. Red asterisks labels caparisons of both VWF-siRNA/DMSO and Luciferase-siRNA/Simvastatin with VWF-siRNA/Simvastatin.

Figure S1

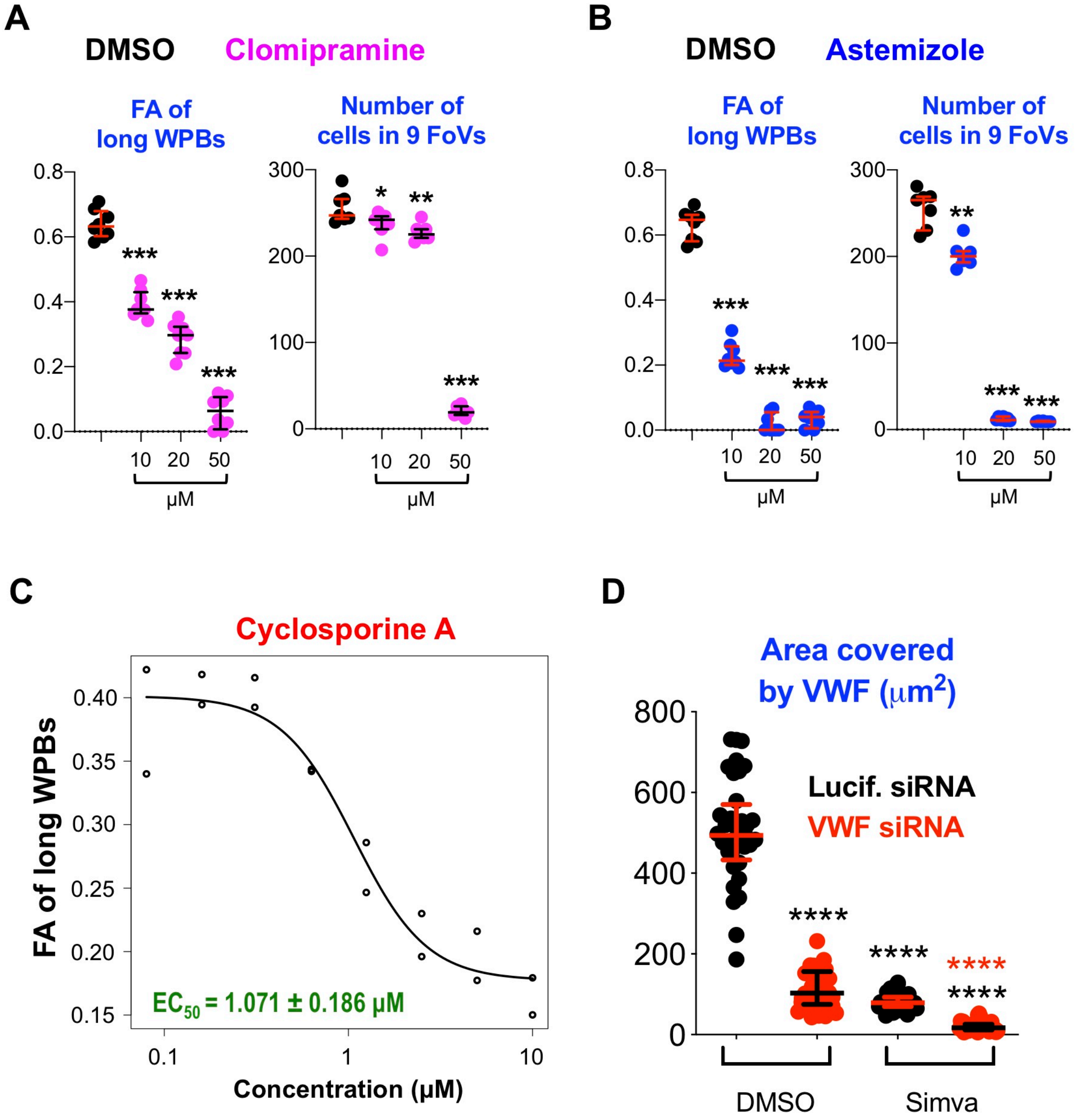

A
DMSO
Clomipramine
FA of long WPBs
Number of cells in 9 FoVs
0.8
0.6
0.4
0.2
0.0
300
200
100
0
***
*
**
10
20
50
μM
B
DMSO
Astemizole
FA of long WPBs
Number of cells in 9 FoVs
C
Cyclosporine A
FA of long WPBs
0.40
0.35
0.30
0.25
0.20
0.15
EC50 = 1.071 ± 0.186 μM
0.1
1
10
Concentration (μM)
D
Area covered by VWF (μm2)
800
600
400
200
0
Lucif. siRNA
VWF siRNA
****
DMSO
Simva
